# The peruvian genome project: expanding the global pool of genome diversity from South America

**DOI:** 10.3389/fgene.2025.1614021

**Published:** 2025-07-23

**Authors:** Heinner Guio, Cesar Sanchez, Victor Borda, Luis Jaramillo-Valverde, Omar Caceres, Carlos Padilla, Omar Trujillo, Julio A. Poterico, Carolina Silva-Carvalho, Mary Horton, Cristina M. Lanata, Alessandra Carnevale, Sandra Romero-Hidalgo, Samuel Canizales-Quinteros, Víctor Acuña-Alonzo, Marco Machacuay-Romero, Pedro Novoa, Roberto Frisancho, Ruth Shady, Pedro Flores-Villanueva, Timothy D. O’Connor, Manuel Corpas, Eduardo Tarazona-Santos

**Affiliations:** ^1^ Centro Nacional de Salud Publica, Instituto Nacional de Salud, Lima, Peru; ^2^ Facultad de Ciencias la Salud, Universidad de Huanuco, Huanuco, Peru; ^3^ INBIOMEDIC Research and Technology Center, Lima, Peru; ^4^ University of Maryland - Institute for Health Computing, University of Maryland School of Medicine, North Bethesda, MD, United States; ^5^ Institute for Genomes Sciences, University of Maryland School of Medicine, Baltimore, MD, United States; ^6^ Departamento de Genética, Ecologia e Evolução, Instituto de Ciências Biológicas, Universidade Federal de Minas Gerais, Belo Horizonte, Brazil; ^7^ National Human Genome Research Institute, National Institutes of Health, Bethesda, MD, United States; ^8^ Laboratorio de Enfermedades Mendelianas, Instituto Nacional de Medicina Genómica (INMEGEN), Mexico City, Mexico; ^9^ Unidad de Genómica de Poblaciones Aplicada a la Salud, Departamento de Biología, Facultad de Química - INMEGEN, Mexico City, Mexico; ^10^ Escuela Nacional de Antropología e Historia (ENAH), Mexico City, Mexico; ^11^Zona Arqueológica Caral, Unidad Ejecutora 003, Ministerio de Cultura del Perú, Lima, Peru; ^12^ Escuela Profesional de Arqueología, Facultad de Ciencias Sociales, Universidad Nacional Mayor de San Marcos, Lima, Peru; ^13^ Department of Anthropology and Center for Human Growth and Development, University of Michigan, Ann Arbor, MI, United States; ^14^ Life Sciences, University of Westminster, London, United Kingdom; ^15^ The Alan Turing Institute, London, United Kingdom; ^16^ Cambridge Precision Medicine Limited, ideaSpace, University of Cambridge Biomedical Innovation Hub, Cambridge, United Kingdom

**Keywords:** ancestry, andes, amazon, Latin America, genomics, global datasets, equity, diversity

## Abstract

The process of inhabiting the Americas by ancestral native American populations involved many individuals settling in the Peruvian Andes and Amazonian regions. Due to Latin American countries representing less than 1% of the human genome data available in public reference databases, the evolution and migration processes involved in adapting have not yet been fully explained. The Peruvian Genome Project is an initiative, started in 2011, to address the genomic data underrepresentation from native South American populations. This project has collected 1,149 samples from 17 traditional native and 13 mestizo (mixed of native, African, and European ancestry) communities. Currently, 150 whole genomes and 873 array-genotyped individuals have been analyzed including coastal, Andes, and Amazonian regions. We discovered 1.6 million novel genetic variants with varying frequencies, indicative of local environmental adaptations and genetic drift. These novel variants allow us to infer adaptive traits and population-specific allele frequencies for people living at different altitudes and varying adaptations to pathogens or living conditions. The Peruvian Genome Project is the result of over a decade of work in sample selection, logistics, and approved regulatory community engagement, designed to enhance the human genome pool of native Americans diversity. The data collected here enable the targeted characterization of endemic diseases, trait adaptations, and new clinical significance variants in South America. The Peruvian Genome Project represents a step forward in international and multidisciplinary efforts to make precision medicine more inclusive and accessible for underrepresented communities in Latin America, offering significant potential for drug development and diagnostics in a neglected continent.

## Introduction

Estimates of native American representation within genome reference datasets currently fluctuate between 1%–5%, depending on the data source (Mills and Rahal, 2020; Corpas et al., 2024). Peru, which encompasses an area of 1,285,215 km^2^, has a population of 33 million inhabitants as of 2023, featuring both mestizos and native peoples, the two ethnic categories broadly used in Latin American societies and census (Salzano and Bortolini, 2001). Historical records indicate a significant demographic shift among its constituent native population, reflecting (in part) a complex social process of urbanization of the Peruvian society (Contrera and s, 2020). In 1620 (almost 90 years after the arrival of the first European), native peoples amounted to 75% of Peru’s population. By 1796, this decreased to 56% and further dwindled to 31% by 2003. Today, according to the Peruvian database of indigenous or native peoples, native populations are fragmented into 55 native communities (Ministry of Culture of Peru, 2024; National Institute of Statistics and Informatics. Peru: Sociodemographic Profile, 2017), including 51 from the Amazon and four from the Andes (Ministry of Culture of Peru, 2024). Many of these populations have been inaccessible for genome and phenotypic characterization. This trend underscores a concerning reality; the proportion of the Peruvian population considered native is declining over time (Dam, 2023). This decline, due to admixing and migration, is both steadily reducing opportunities for exploring the original genetic landscape of the Americas and decreasing chances for charting endemic evolutionary adaptations specific to a unique set to climates and environments (Salzano and Bortolini, 2001). Therefore, the observed decline in the population classified as native Peruvians translates into a continual reduction in the prospects for investigating novel genetic variants harbored by these communities. It is thus critical for the scientific community to act now and ethically engage with these understudied communities before the current window of opportunity disappears.

Approximately 30% of the population of Peru resides in the high elevation Andes above 2,500 m above sea level (m.a.s.l.) (Tremblay and Ainslie, 2021). The Andes and the Amazon jungle have influenced the environmental exposure of local populations. The population structure within the Western South America region exhibited a gene diversity of 0.489 (95% CI: 0.448–0.525) among Andean populations, based on Y-chromosome marker analyses (Tarazona-Santos et al., 2001). This level of genetic diversity suggests historical patterns of intra-regional migration within the Andes, promoting gene flow among highland communities. This pattern of migration has likely conditioned populations to remain in their environment, fostering diverse adaptive changes such as response to hypoxia (Xing et al., 2008). The underlying genetic mechanisms for local adaptations and their interactions with environmental factors, lifestyle, and potential epigenomic modifications remain to be fully elucidated. These factors collectively contribute to physiological, endocrine, cardiovascular, respiratory, and other systemic changes observed in native populations (Bigham et al., 2010; Beall, 2000).

**FIGURE 1:**
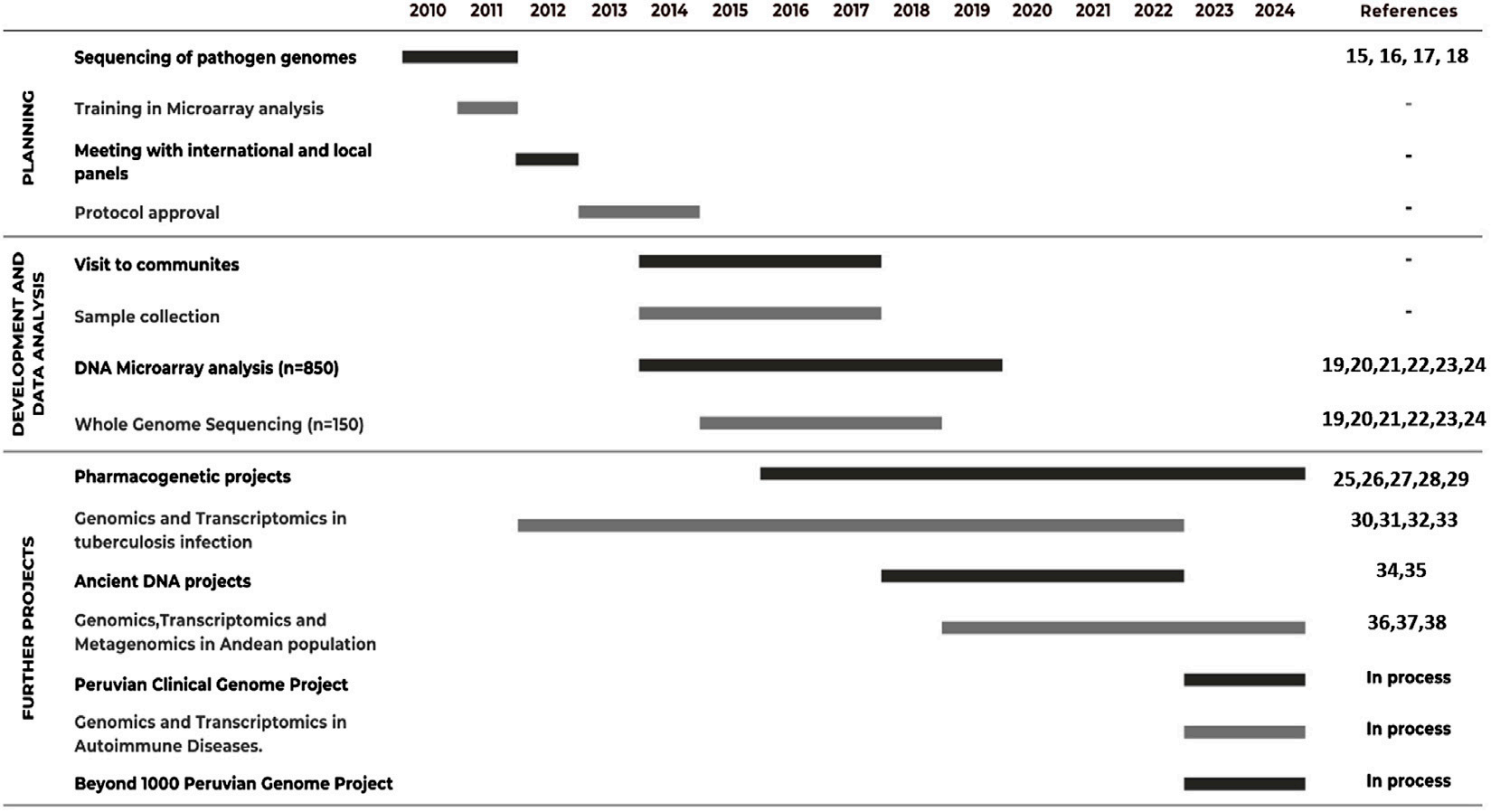
Timeline for the development of the Peruvian genome project and future initiatives.

When carrying out the analysis of coastal, Andean, and Amazonian populations, it is useful to subdivide them according to their proportion of admixture. The examination of both mestizo and native population classification offers a valuable framework for advancing genomic medicine in Peru and the broader Americas (Tishkoff and Verrelli, 2003; Jorde et al., 2001). This has the potential to identify health disparities according to the prevalence of diseases in these communities (e.g., Acute Respiratory Infections, Cerebrovascular Diseases, Ischemic Heart Diseases, Hypertensive Diseases, Septicemia, Cirrhosis, Injuries of Undetermined Intent, Diabetes Mellitus, Stomach Cancer and Kidney Failure) (Tarazona et al., 2014). These project findings, as illustrated in Figure 1, pave the way for further advancements toward precision medicine and data equity within a dramatically underrepresented set of human populations in the Latin American region.

The initial stages of the Peruvian Genome Project (PGP) involved the sequencing of pathogen genomics in 2010. Since then, the project evolved into a tailored-made protocol for selecting populations and individuals for sequencing and genotyping from across the diverse geography of Western South America. This protocol involved international experts and local authority panels, starting from 2012. In 2013, we started visiting communities with which we engaged via a variety of channels, including local media, presentations, and interviews. These interviews included chiefs (“Apus”) from local communities as our initial point of contact and exchange of information. Once appropriate consent from Apus and authorized individuals were obtained, sample collection, sequencing, and genotyping started in 2014 and went through to 2017. The sequencing was performed at the New York Genome Center (NYGC) and the array genotyping at the facility of the Instituto Nacional de Salud del Peru (the Peruvian National Institute of Health). This culminated in 2018 when we showed that the studied populations of the three geographic regions in Peru (Amazon, Andes, and coast) diverged from each other ∼12,000 ya (Harris et al., 2018). Further research allowed us to shed light on the susceptibilities highlanders have in tuberculosis infections and their related pharmacological responses (Jaramillo-Valverde et al., 2024; Ganachari et al., 2012). During the COVID pandemic (2020–2022), we shifted our focus to collaborating with international research groups to incorporate ancient Peruvian DNA samples. Since then, we have focused our efforts on developing a repository of genome and phenome data that goes beyond the current ∼1,000 genomes collected to date. As we continue exploring the wealth of diversity that the Andes, Amazon, and coast accumulate in Western South America, we have worked with special emphasis on the endemic diseases and precision medicine approaches that apply to local native populations.

## Population selection criteria and sample collection strategies

**FIGURE 2:**
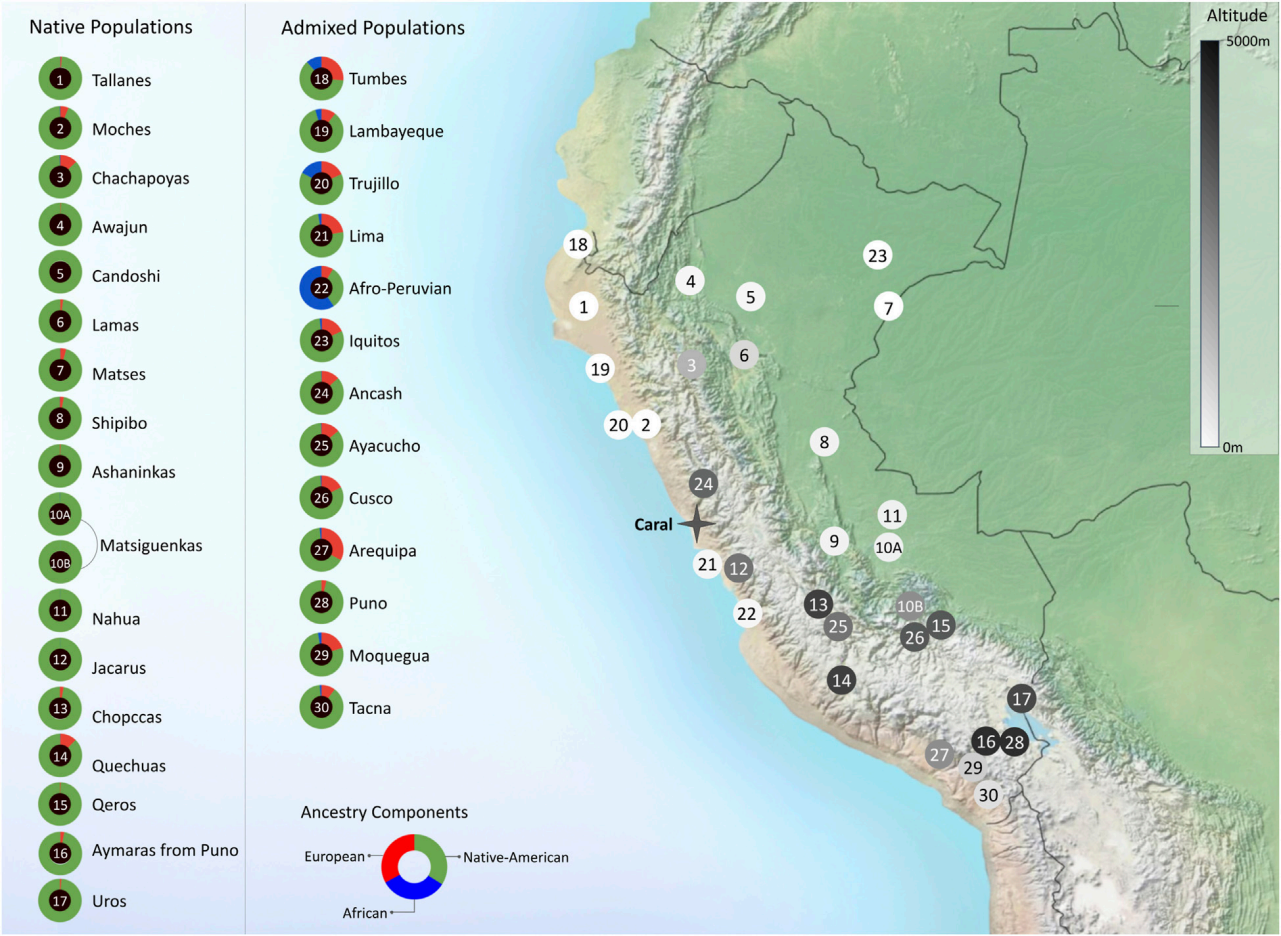
Genome-wide ancestry proportions and geographic distribution of Peruvian populations selected for the Peruvian Genome Project. Doughnut plots show the degree of admixture for each population, inferred by the mean of the result of K = 3 from the ADMIXTURE software (Alexander et al., 2009). The ancestry components of African, European, and Native American origins are represented within these doughnuts in blue, red, and green, respectively. An adjacent map shows the location of the different populations in numbered circles shaded with different intensities to indicate the average altitude at which the population usually resides. Shading corresponds to altitude bins: light (low altitude <500 m), medium (500–2,000 m), and dark (high altitude >2,000 m).

**FIGURE 3:**
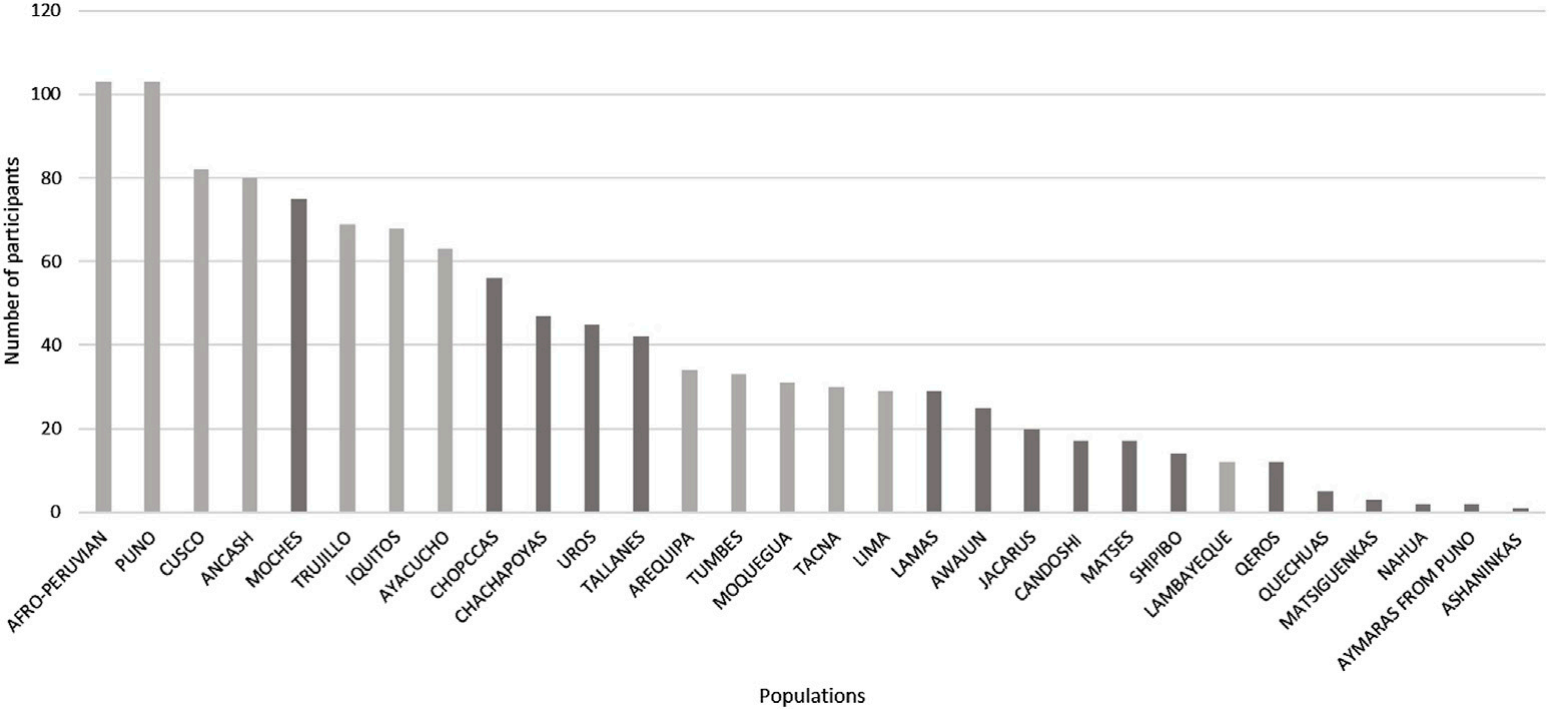
Total number of participants recruited for the Peruvian Genome Project (n = 1,149) and the breakdown of communities from which they come from black: native populations, grey: admixed population. In some communities was difficult to get more than one sample from individuals who had parents and grandparents form the same native communities, show in as than the process of admixture/migration is diluting the native component.

The presence of the Andes, which cross North to South the South American continent (Figure 2), has dramatically shaped human evolution and adaptation in the region. Nearly 30% of the Peruvian population lives above 2,500 m above sea level on the Andes, whereas 10% live in the Amazon jungle, East side of the mountains. These landscapes exhibit different pathogenic and isolating environmental conditions (Accinelli and Leon-Abarca, 2020). To carry out a balanced recruitment and identification of population diversity criteria by the mandate of the Instituto de Salud Nacional del Perú, we convened a local and an international research panel as follows. 1) A Local Panel composed of representatives of the native communities of Peru, the Ministerio de Cultura, Non-Governmental Organizations, archaeologists, sociologists, and anthropologists. This panel identified three criteria for inclusion: representativeness (number of residents in each population); degree of isolation; and vulnerability to disappear by admixture/cultural absorption with other populations, displacement by migrations, or difficult access. Using these criteria, 17 native populations and 13 mestizo populations were identified, spanning diverse locations and geographical distances (Figure 2). Figure 3 shows the breakdown of all 1,149 recruited community participants. Additionally, for participant selection, we considered historical migratory routes and self-reported ancestry to better trace patterns of movement across regions. These factors helped us capture the effects of admixture and the preservation of cultural and demographic heritage within communities. Because of this, we defined “native individuals” those whose parents and grandparents were born in the same native community. This criterion made it difficult to get more than one sample from individuals who had parents and grandparents from the same native communities. 2) An International Panel was composed of researchers who had already developed projects in similarly underrepresented countries, such as researchers from INMEGEN (National Institute of Genomic Medicine of Mexico), the University of Michigan (United States), University of Maryland (United States), and Universidade Federal de Minas Gerais (Brazil). These researchers’ experience allowed us to consider appropriate clinical measurements and analyses not necessarily considered in previously peer-reviewed projects.

## Ethical approval process and cascade of consents

The ethical process for collection, stewardship, and dissemination of data and results for gathered samples was meticulously implemented and carried out at the community and individual levels. We ensured that communities and individuals were engaged according to international and local protocols, including the Declaration of Helsinki for medical research involving human subjects. For community participation, a consultation process was performed involving authorities at the national level (Ministerio de Salud, Peruvian Government), regional authorities, and several Peruvian universities. The information gathering strategy for sample collection began a month in advance for each community, including communication materials. These materials consisted of: i) the distribution of a brochure that explains the project written in simple language (Spanish or local native language); ii) the display of a poster that reproduces the informed consent format; iii) public informative sessions aimed at the participating community; and iv) communication through local television, radio, and written press whenever possible or available. Native communities were visited several months before sampling to request authorization from the community leaders (Apus). The final decision for participation was made by the individuals themselves who had to understand and consent to the ethical processes outlined here. Subjects that matched the inclusion criteria were contacted to participate, and informed consent and authorization to preserve their samples were obtained. All participants were able to withdraw from this study at any time with no explanation required. All participants gave informed consent in the presence of a translator to their mother-tongue traditional language and two local witnesses. Twenty-two individuals that met the inclusion criteria did not participate due to religious/cultural beliefs, and two of the subjects who were recruited left the study by requesting to delete their samples and data. All procedures were reported and presented for evaluation and approval by the Research and Ethics Committee from the Instituto Nacional de Salud del Peru (authorization no. OI-003-11 and no. OI-087-13).

## Data sampling and genotyping

Participants were selected to represent diverse self-described Native and Mestizo Peruvian populations. We applied three criteria to optimize participant selection to best represent the Indigenous American populations. These included: (i) the place of birth of the participant, his or her parents and grandparents (they all had to belong to the same community), (ii) their last names (selecting only those corresponding to the region if they existed or were known), and (iii) if several members of a family met our standards for inclusion, the oldest member was then selected for our study.

The first phase of this study included 150 Native and Mestizo Peruvian whole genomes sequenced to an average of 35X coverage on an Illumina HiSeq X 10 platform by the New York Genome Center (NYGC). An additional 873 Native American and mestizo Peruvian individuals were genotyped using a 2.5 M Illumina chip, featuring over two- million markers for dense genome-wide coverage and extensive disease-associated content at the Biotechnology and Molecular Biology Lab of the “Instituto Nacional de Salud del Perú”.

## Sample size bias and weighted pca correction

To assess and mitigate potential biases introduced by unequal sample sizes across Peruvian populations, we performed a comprehensive analysis of the distribution of sample sizes by population, ancestry group, and geographic region. We implemented a weighted principal component analysis (PCA), where weights were assigned inversely proportional to group sample sizes, and conducted stratified bootstrap resampling to ensure robustness in population structure inference (Supplementary Figures S1, S2), which summarize population representation. Sample classification was based on a harmonized population mapping file curated from sequencing and genotyping identifiers. Groupings were further categorized by ancestry and geography, and matching was validated using regex-enhanced sample ID extraction routines.

## SNP chip ascertainment bias evaluation

To evaluate the extent of SNP chip ascertainment bias arising from the use of a European-designed array, we compared variant representation and allele frequency distributions between genotyping data (2,040,576 SNPs, 722 individuals after identify by descent (IBD) pruning) and whole genome sequencing (WGS) data (12,897,474 variants, 109 individuals after IBD pruning) from comparable Native American populations. We assessed genomic capture rates, frequency spectrum distortion, and chromosomal bias using PLINK-derived variant statistics and minor allele frequency bins. Overall, only 11.0% of WGS variants were captured by the SNP array, with rare variants underrepresented by 11.6 percentage points and common variants overrepresented by 6.4 percentage points (see Supplementary Figures S3–S5).

## Variant validation

Variant-level quality was evaluated using summary statistics and bcftools-derived metrics. We assessed transition/transversion (Ts/Tv) ratios, variant burden, sample size, and multiallelic site rates to validate the 12.9 million detected SNPs. The observed Ts/Tv ratio of 2.09 falls within the expected high-quality range (2.0–2.2). Conservative error modeling estimated that only 0.59% of variants may represent artifacts, implying 99.4% of variants are high-confidence. A multi-metric validation framework incorporating population-scale allele frequency consistency, Hardy-Weinberg equilibrium, and variant density patterns further supports the authenticity of these calls (Supplementary Figure S6).

## Quality control metrics

We performed standard quality control (QC) analyses for both SNP array and WGS datasets, assessing sample and variant call rates, heterozygosity, minor allele frequency (MAF), and Hardy-Weinberg equilibrium (HWE). All samples exceeded the 95% call rate threshold, and mean heterozygosity and MAF distributions aligned with expected human genome profiles. QC thresholds applied included ≥95% call rate, MAF ≥1% for common variants, and HWE p ≥ 1 × 10^−6^. Summary metrics for both platforms confirm high-quality genotyping and sequencing data (Supplementary Figure S7).

## Phenotype data and laboratory measurements

To date, we have collected a total of 1,149 samples (each corresponding to a different individual) from 17 traditional Native and 13 Mestizo (admixed of Native Peruvian, African, Asian, and European ancestry) communities.

Demographic information, including lifestyle (smoking status and diet patterns) and anthropometrics data (such as body mass index (BMI), weight, and height), were collected. Blood samples were obtained in fasting conditions to encompass blood lipids, blood cell traits (mean hemoglobin levels, red cell count, white cell count, and platelets), glucose levels, and renal function markers (Table 1).

**TABLE 1:** Characteristics of participants enrolled in the Peruvian Genome Project (PGP).

Characteristic	All (n = 1,149)	Native (n = 506)	Mestizo (n = 643)
Number of individuals interviewed, N	1149	506	643
Gender N (%)			
Female	382 (33.3)	185 (36.6)	197 (30.6)
Male	767 (66.7)	321 (63.4)	446 (69.4)
Location			
North	229 (22.8)	114 (24.5)	115 (24.5)
South	194 (19.3)	16 (3.4)	178 (38.0)
Center	354 (35.3)	178 (38.2)	176 (37.5)
East	226 (22.6)	158 (33.9)	0 (0.0)
Age (years), median (IQR)	41 (23–56)	53 (38–68)	32 (21–41)
Age group (years), N (%)			
18–19	98 (8.7)	7 (1.4)	91 (14.2)
20–29	363 (32.3)	60 (12.3)	303 (47.4)
30–39	142 (12.6)	68 (14.0)	74 (11.6)
40–49	163 (14.5)	90 (18.5)	73 (11.4)
50–59	142 (12.6)	87 (17.9)	55 (8.6)
60+	217 (19.3)	174 (35.8)	43 (6.7)
Body mass index (kg/m2), mean ± SD	25.4 ± 5.58	26.0 ± 5.19	24.9 ± 5.83
BMI classification, N (%)			
Underweight	25 (2)	12 (2)	13 (2)
Normal	516 (45)	198 (39)	318 (50)
Overweight	423 (37)	198 (39)	255 (35)
Obese	177 (16)	96 (20)	81 (13)
Smoking status, N (%)			
Current smoker	139 (12.1)	64 (12.7)	75 (11.7)
Never smoked	1006 (87.9)	441 (87.3)	565 (88.3)
Alcohol consumption, N (%)			
Never or no alcohol use	647 (56.8)	306 (60.8)	341 (53.6)
Frequent current drinker	493 (43.2)	197 (39.2)	295 (46.4)
Metabolic markers (mean ± SD)			
Total cholesterol (mg/dL), mean ± SD	163.1 ± 48.18	169.0 ± 50.83	159.4 ± 46.08
High-density lipoprotein (mg/dL), mean ± SD	53.0 ± 19.06	55.5 ± 18.55	49.0 ± 19.20
Low-density lipoprotein (mg/dL), mean ± SD	85.7 ± 50.14	93.8 ± 53.17	80.7 ± 47.51
Triglycerides (mmol/L), mean ± SD	127.8 ± 83.59	138.7 ± 88.15	120.9 ± 79.89
Bilirubin, median (IQR)	0.8 (0.56–1.30)	1.0 (0.72–1.6)	0.8 (0.48–1.11)
Blood Pressure			
Systolic pressure (mmHg), mean ± SD	111.9 ± 18.5	115.5 ± 22.8	109.6 ± 14.7
Diastolic pressure (mmHg), mean ± SD	69.7 ± 48.18	69.9 ± 12.3	69.5 ± 10.0
Anthropometric Measurements			
Weight (kg), mean ± SD	63.7 ± 14.61	63.2 ± 14.26	64.1 ± 14.88
Height (cm), mean ± SD	157.3 ± 9.09	155.0 ± 9.30	159.1 ± 8.49
Anemia			
White blood cell count	8.1 ± 4.51	8.1 ± 5.46	8.1 ± 3.81
Red blood cell count	45.3 ± 13.77	43.6 ± 6.95	46.4 ± 16.59
Hemoglobin	14.9 ± 2.09	14.4 ± 2.33	15.18 ± 1.87
Platelet count (PLT)	316.9 ± 76.94	296.4 ± 81.09	329.6 ± 71.40
Lymphocytes	29.3 ± 11.80	28.2 ± 14.14	29.9 ± 10.02
Monocytes	4.1 ± 2.94	4.4 ± 3.20	3.9 ± 2.75
Basophils	0.2 ± 0.61	0.2 ± 0.59	0.3 ± 0.62
Eosinophils	2.7 ± 3.72	3.6 ± 5.05	2.2 ± 2.42

We were able to infer significant differences between Native and Mestizo samples due to environmental factors such as geographical altitude and diet. BMI and ancestry, which have been associated with specific environmental conditions in previously related studies (Deurenberg et al., 2002), showed significant differences in BMI between Native and Mestizo populations. However, more precise methodologies need to be considered in future studies in order to discriminate whether these differences are related to excess fat or muscle mass percentage.

## Initial findings and key contributions for genomic medicine in peru and native americans

The PGP offers significant insights into the genetic diversity and evolutionary adaptations of Native and Mestizo populations in Peru. This work is being applied to a range of clinical interventions that are actionable for the advancement of precision medicine, public health strategies, and understanding of human genetic evolution (Guimarães Alves et al., 2021). In what follows, we present some examples that illustrate how our work has shed light on our understanding of native Peruvian genome variants endemic from these populations.

### Population structure

Of the 26 populations analyzed, 15 met the recommended minimum of 30 samples per group for stable allele frequency estimation and PCA inference. Eleven populations (42%) fell below this threshold, and four populations had fewer than 10 samples (e.g., NAHUA, QEROS). This yielded a sample size imbalance with an 8:1 ratio between the largest and smallest populations. To minimize distortion in PCA projections and allele frequency estimates, we implemented weighted PCA, stratified bootstrap resampling, and grouped analysis of related populations. The resulting structure remained consistent across resampling, indicating robustness of the inferred clusters (see Supplementary Figures S1, S2).

Comparative analysis revealed substantial bias in variant representation on the SNP chip relative to WGS. The array captured only 11.0% of variants present in the WGS dataset. Allele frequency distributions were skewed, with rare variants (<0.5% MAF) being underrepresented by 11.6 percentage points and common variants (>10% MAF) overrepresented by 6.4 points. This ascertainment bias likely inflates F_ST_ values and distorts ancestry inference, especially in populations with high Native American ancestry. These distortions are visualized in the multi-panel summary (Supplementary Figure S3) and in targeted views of allele frequency (Supplementary Figure S4) and capture rate (Supplementary Figure S5).

### Variant-level quality metrics support data reliability

Among the 12.9 million variants identified across 150 individuals, we observed a Ts/Tv ratio of 2.09 and ∼85,000 variants per individual, metrics aligned with known human WGS benchmarks. Conservative error modeling yielded a 0.59% upper-bound error rate. These results support the authenticity of >99% of variant calls, minimizing concerns about false positives. Validation figures are shown in Supplementary Figure S6. Both the SNP array and WGS datasets demonstrated high call rates (>99%) and typical MAF distributions. The array dataset captured 2.0 M variants, while the WGS dataset covered 12.9 M SNPs. Despite having no overlapping samples, both datasets passed standard QC thresholds, with <5% of variants violating HWE and call rates above 95% for >99% of data points. These QC outcomes support the validity of using both datasets for population and pharmacogenomic analyses.

### Enhanced understanding of genetic diversity and disease susceptibility

It has been previously shown that the Peruvian Mestizo populations have at least a 60% genetic Indigenous American ancestry, with some native communities adding up to 90% of their genetic native component as shown in Figure 2. We have discovered 1.6 million novel genetic variants according to the Variant Effect Predictor (VEP) (https://grch37.ensembl.org/Homo_sapiens/Tools/VEP), which are not present in existing data banks resources such as dbSNP (Jorde et al., 2001). The analysis of high-impact mutations, as classified by the VEP, revealed significant insights into potential pathogenic variants within the genomes of Peru’s indigenous and mestizo populations. Out of 1,235 high-impact mutations identified in protein-coding regions, notable categories included premature stop codons (597 variants), which often lead to nonfunctional proteins, and splice site mutations (322 variants) that can disrupt gene expression by altering mRNA splicing. Start codon losses (58 variants) and stop codon losses (26 variants) were also observed. These can severely affect protein synthesis, resulting in absent or aberrant proteins. Clinically, 22 variants were classified as pathogenic or likely pathogenic, indicating their relevance to known disease mechanisms. Others were deemed benign (39 variants) or of uncertain significance (7 variants), emphasizing the complexity of variant interpretation. Notably, 928 of these high-impact variants had no classification in existing databases, underscoring the distinctive genetic profile of the Peruvian population and the need for ongoing research.

The inclusion of variants from under-represented populations such as Native Peruvians in global genomic databases is expected to help refine and broaden the accuracy of genetic risk assessments and pharmacogenomics interventions for diseases most prevalent for indigenous American populations, whose pharmacogenomic representation is only 0.1% of existing data (Corpas et al., 2024). These efforts may lead to more personalized and effective healthcare interventions. It also investigates the effects of South American genetic diversity on traits and disease risk, which will benefit any individual carrying these variants, whatever their proportion of Native American ancestry.

One of the initiatives preceding the Peruvian Genome Project was the 1000 Genomes Project (1000 Genomes Project Consortium et al., 2015), which included 85 genomes of Peruvians from Lima. These individuals, although helpful, were all sampled from Mestizo individuals from Lima, which means they have more European ancestry and were not representative of the diversity richness of Native communities in Peru. The advantage of the PGP lies in the sophisticated inclusion criteria carried out to consider different biogeographical regions from across the coast, Andes, and Amazon. This allowed us to evaluate the effects of migration over thousands of years as well as helping differentiate populations based on population bottlenecks, revealing a distinct genetic fingerprint on the Americans’ ancestors (Yang et al., 2021). Moreover, we have found that the degree of ancestry (mestizos vs. natives) and the geographic altitude habitat (high vs. low landers) are linked (Jaramillo-Valverde et al., 2024; Jaramillo-Valverde et al., 2023).

Unique genomic changes in the composition of Peruvian populations, such as the ones we are beginning to uncover, have aided in elucidating the impact of ancient migrations, helping determine population structure based on geographic barriers in Peru (Jaramillo-Valverde et al., 2023; Nakatsuka et al., 2020; Arango-Isaza et al., 2023). In South America, genetic studies have discovered a substantial differentiation between the Andes and Amazonia, which has been framed within a model of large communities connected by gene-flow in the Andes versus small, isolated communities in Amazonia (Tarazona-Santos et al., 2001; Fuselli et al., 2003; Barbieri et al., 2014; Barbieri et al., 2019). Our data provides evidence of migrations in the central and northern Andean and Amazon regions, which are restricted in the Southern Andes and are likely due to the effect of the high elevation of the Andean mountains. Our findings for the metabolism of antituberculosis drugs and immune response show differences according to the location and geographic altitude of the populations (Jaramillo-Valverde et al., 2024; Jaramillo-Valverde et al., 2023).

### Pharmacogenomics and drug safety

Genetic ancestry plays a central role in population pharmacogenomics (Yang et al., 2021). In one of our studies, we researched the presence of adverse reactions during anti-tuberculosis treatment in the Peruvian population. Our results suggest that 30% of the Peruvian populations are associated with the slow metabolism of isoniazid (Levano et al., 2021). We also identified haplotypes with divergent associations with drug-induced liver injury (DILI), based on the mestizo or native Peruvian population. For instance, we found evidence of NAT2*5B and NAT2*7B being associated with DILI risk in mestizos, while no such association has been observed in natives. Additionally, haplotypes NAT2*5G and NAT2*13A have only been negatively associated with DILI in the studied Native Peruvians (Jaramillo-Valverde et al., 2024). Current research also suggests a greater prevalence of probable hepatotoxicity in the Amazonian population. In a study still in progress, we have compared the pharmacogenetic response to antituberculosis drugs in a population with ancestry greater than 95% native from the coast, Andes, and the Amazon of Peru. Our initial results point to greater hepatotoxicity in antiretroviral cotreatment on the coast. This is one example of the importance of conducting more human genomics studies on underrepresented populations (Harris et al., 2018); it aids the identification of genetic variants clinically relevant in pharmacogenetics (levels 1 and 2 of the PharmGKB database) that are different between Andean and Amazonian populations. Thus, by considering the genetic diversity within and between populations, healthcare providers are beginning to better predict adverse drug reactions, adjust dosages, and select the most appropriate medication, thereby enhancing patient safety and treatment efficacy (Jaramillo-Valverde et al., 2022a; Jaramillo-Valverde et al., 2022b).

### Adaptation to high altitude and its health implications

PGP’s findings on the selection of genes related to immune response in different Peruvian populations offer promising data for understanding susceptibility and resistance to infectious diseases. We revealed that environmental and genetic differentiation between Andean and Amazonian populations has shaped immune-related genetic variation through natural selection and drift. This includes genes associated with immune function (e.g., *CD45*, *DUOX2*), thyroid function (*DUOX2*), cardiovascular traits (*HAND2-AS1*), hematological pathways (*TMPRSS6*), and drug response genes (Borda et al., 2020). Considering that genetic drift and natural selection have exacerbated genetic differences among local populations, our group embarked on finding how this effect may be manifested in gene expression.

In previous studies, the immune response has been mainly associated with ancestry. However, our findings show that altitude and the microbiome are also as important as ancestry in the immune response in populations with a high Indigenous American ancestry. To evaluate the immune response in Native Highlanders, a laboratory was developed at the University of Huanuco, located at 3,000 m above sea level, to carry out a transcriptomic project resulting from the stimulation of PBMCs with proteins from bacteria, viruses, and fungi. For this aim, we intended to address the research question of how the expression of immune response-associated genes varies in high-altitude populations compared to those not inhabiting such geographic conditions. Our data suggest that there is indeed a difference in the immune response of these native inhabitants, with upregulated expression in genes such as *HLA-DPB1, FN1, CD36, MMP2, HLA-DRB1, FCGR1A, CCL17,* and *HLA-DRB5* and downregulation in *TGAX, CCL22, CSF1, CXCL8, IL12A, MMP9, CSF2, PTGS2*, and *FGF2*. We reported the expression of these genes from individuals with more that 60% of native ancestry in the central Peruvian highland region (Jaramillo-Valverde et al., 2023). The gene expression of the genes in our study do not report a change in expression in the immune response of Africans and Europeans using the same methodology (Quach et al., 2016). While these gene expression data provide strong support for environmental and ancestral contributions to immune modulation at high altitude, we acknowledge the absence of matched physiological phenotypes (e.g., oxygen saturation, hemoglobin levels). Therefore, our interpretation of high-altitude adaptation remains provisional and focused on molecular correlates rather than definitive trait-level adaptation. Nonetheless, these results suggest that ancestry and local environments could influence the immune response in humans as showed by Randolph et al. (2021).

### Prevalence of established autoimmune risk variants in the PGP

Autoimmune diseases, in particular systemic lupus erythematosus (SLE), tend to manifest at a younger age and with worse clinical outcomes in people of non-European descent. Immune-related genetic variants appear to evolve differently depending on the geographic distribution and intensity of pathogen exposure. In fact, nearly 13% of non-HLA genome-wide association study (GWAS) loci associated with systemic lupus erythematosus (SLE) show signatures of natural selection, suggesting adaptation to local infectious pressures (Wang et al., 2021).

We studied 670 individuals from the PGP, 2068 individuals from the 1000 Genomes Project Phase 3 release, and 47 patients with SLE from Lima, Peru. Ancestry was inferred using Admixture and RFmix. Data were imputed with the TOPMed Imputation server and annotated to hg38. We compared the frequencies of 199 SLE-associated risk variants among study participants. We also calculated SLE genetic risk scores (GRS) and F_ST_ statistics. All 199 SLE risk SNPs had highly significant differences in frequencies across Peruvian and other continental populations (p values < 0.001). Indigenous Peruvians have higher polygenic risk for SLE compared to European, African, South Asian, and East Asians. FST analysis of SLE risk variants revealed the largest FST between Peruvians and Africans (0.12), and the smallest between Peruvians and East Asians (0.09). Further, it is noteworthy that healthy Native Peruvians exhibit elevated unweighted polygenic risk for SLE in contrast to European, African, and South Asian counterparts, aligning more closely with East Asians. Confirmation of these findings necessitates comprehensive population-based studies within the Americas. These findings emphasize the need for continued genetic investigations into autoimmunity in Peru, moving beyond Eurocentric genetic frameworks, which may offer insights into the heterogeneity of SLE.

## Limitations

Scientific discoveries are starting to emerge from the Peruvian Genome Project (PGP), but it is essential to acknowledge its limitations, which mostly relate to its modest size. Peru’s limited participation in genomic research is due to a confluence of factors such as the country’s lack of prioritization of genomic medicine policies, a shortage of adequately trained genomic scientists, and the centralized research infrastructure in the capital city. We acknowledge that unequal population sample sizes can skew allele frequency estimates, reduce statistical power, and disproportionately influence PCA clustering. Although most major populations were adequately represented, 11 of 26 populations were underpowered (<30 individuals), with four falling below 10. To address this, we implemented weighted PCA and performed stratified bootstrap analyses to reduce bias and quantify the extent of distortion. These approaches yielded consistent ancestry inferences, supporting the validity of our conclusions. Nonetheless, results involving underpowered groups (e.g., NAHUA, QEROS, MATSIGUENKAS) should be interpreted with caution, and hierarchical grouping or confidence intervals are recommended for future analyses.

Further, the use of a genotyping array designed with European reference populations introduces systematic ascertainment bias. This is evident from the 89.0% loss of variation compared to WGS and disproportionate representation of common variants. Such biases likely inflate observed genetic differentiation and obscure fine-scale structure in Native American populations. While the principal axes of variation appear robust, subtle signals of substructure and admixture should be interpreted with caution. Incorporating WGS or imputed datasets with ancestry-matched reference panels would help mitigate these limitations.

## Next steps

### Ancient DNA studies in samples from 5000 years ago

The genetics of present-day Peruvian native populations largely originates from the ancestral diversity of Early Native Americans and accumulated mutations since their initial settlement. While admixture, primarily with European colonizers and to a lesser extent with more recent migrants, has contributed to their genetic composition, ancient DNA studies are crucial for unraveling their pre-Columbian history. Research in the Andes has revealed complex population dynamics, demonstrating both genetic continuities, as well as significant population movements, gene flow, and notable ancestry diversity. Additionally, genetic adaptations to environmental factors such as diet and disease pressures after European contact have been identified, as well as high-altitude adaptations. Despite these advancements, the precise origin of many genetic variants in ancient Peruvian populations, and whether these were introduced by early migrations or arose locally, still presents significant knowledge gaps due to the scarcity of samples from certain periods and areas. Although ancient DNA analyses of human remains recovered from Caral (Figure 2), the oldest civilization in the Americas, have not yielded conclusive results, ancient DNA has been successfully recovered from the microbiome preserved in archaeofeces. This material provides unique insights into microbial diversity, dietary habits, agricultural practices, seasonal migration, and the health status of Caral’s inhabitants. To this end, a mobile laboratory has been implemented in the Supe-Caral valley to rapidly extract DNA and minimize contemporary contamination (Guio et al., 2022). This methodology has been validated and utilized at archaeological sites in France (Utge et al., 2020). While this initiative is ongoing, we acknowledge that comparisons between present-day and ancient genomes must control for post-Columbian admixture, bottlenecks, and low-coverage artefacts. We therefore interpret any ancient–modern similarities cautiously and plan to incorporate admixture-aware models in downstream analyses. We also recognize the foundational contributions of prior Andean aDNA studies (e.g., Nakatsuka et al., 2020; Skoglund et al., 2015; Arango-Isaza et al., 2023), which provide valuable comparative frameworks for testing our hypotheses.

### Peruvian clinical genome

Previously, we found genetic variants associated with the prognosis, severity, and treatment response in tuberculosis infection (Jaramillo-Valverde et al., 2022b). As the pathogenic potential of newly discovered genetic variants within the PGP remains uncertain, a collaborative effort with the University of Westminster is underway to establish a comprehensive database of the Peruvian clinical genome. This database aims to enable and streamline specialized medical studies, elucidating the clinical significance of these variants in relation to Peru’s most prevalent diseases.

### Genomics and transcriptomic in cardiovascular-related genes in highlanders

All genes are not expressed at all times in all tissues. Considering the new genetic variants associated with cardiovascular health found in the PGP, we decided to combine genomic and transcriptomic analysis of cardiovascular genes to better understand their expression in response to high altitude. This effort is in collaboration with Queen Mary University of London.

### Beyond 1000 peruvian genome project (B1000PGP)

The rigorous data collection methodology adopted by PGP was developed in concordance with the *a priori* questions posed by an international panel of experts from related projects, specifically regarding opportunities and variables relevant to include in the PGP framework. We are currently deliberating the inception of B1000PGP, a project aiming to collect a broader set of variables beyond genomic data, incorporating additional samples progressively over time. We are conducting assessments on both healthy individuals and those diagnosed with various conditions. This effort encompasses not only the elucidation of the spectrum, prevalence, and clinical implications of germline and tumor genomic variants, but also extends to emerging areas such as the microbiome and DNA methylation, recognizing their critical roles as health markers. To that end, we are actively developing initiatives to generate datasets that integrate these additional layers of biological information.

## Conclusion

Peru’s limited engagement in genomic research stems from a confluence of factors. Importantly, the nation’s lack of prioritization of genomic medicine policies leads to a lack of strategic direction and investment. Additionally, the scarcity of adequately trained genomic scientists impedes the development of a proficient workforce capable of advancing research in this field. Meanwhile, centralized research infrastructure in the capital city restricts access and opportunities for researchers in the countryside. Cumbersome administrative processes further hamper Peru’s ability to undertake large-scale genomic studies. Nevertheless, the PGP has managed to implement a multidisciplinary approach throughout years of work and international collaborations, establishing rigorous criteria for the selection and definition of mestizo and native populations. Ethical considerations and stringent methodologies, alongside strict inclusion criteria, have facilitated the identification of participants with substantial Indigenous American ancestry. This has yielding the discovery 1.6 million novel genetic variants pertinent to understanding migration, adaptation, and immune response in native Americans and highlanders. These findings show great promise for potential clinical applications. To propel genomic research forward in Peru, it is imperative to foster international collaborations, particularly through training grants for doctoral and post-doctoral positions. Equally crucial is the development of infrastructure conducive to initiating new projects and facilitating the interpretation of results. Such multidisciplinary collaborative efforts are indispensable for elucidating the genomic heritage of Peruvian populations residing in the Andean and Amazonian regions, thereby enriching our collective understanding of humankind.

## Data Availability

The data presented in the study are deposited in the European Genome-phenome Archive (EGA) repository, (Whole genome sequences from 150 individuals (accession numbers: EGAD00010001958, EGAD00010001990). Genotypic data from 873 array-genotyped individuals (accession numbers: EGAD00010001991, EGAD00010001992)).
