# Supplementary material for: The peruvian genome project: expanding the global pool of genome diversity from South America
Source: Front Genet. 2025 Jul 23;16:1614021. doi: 10.3389/fgene.2025.1614021 (PMC12325035; doi:10.3389/fgene.2025.1614021)
Supplement: Supplementary file 1 [file DataSheet1.docx]

# Supplementary Materials

# Sample Size Bias

This section presents sample size analysis across Peruvian populations. It includes visualizations summarizing group size distribution and implications for population structure inference.

## Supplementary Figure S1


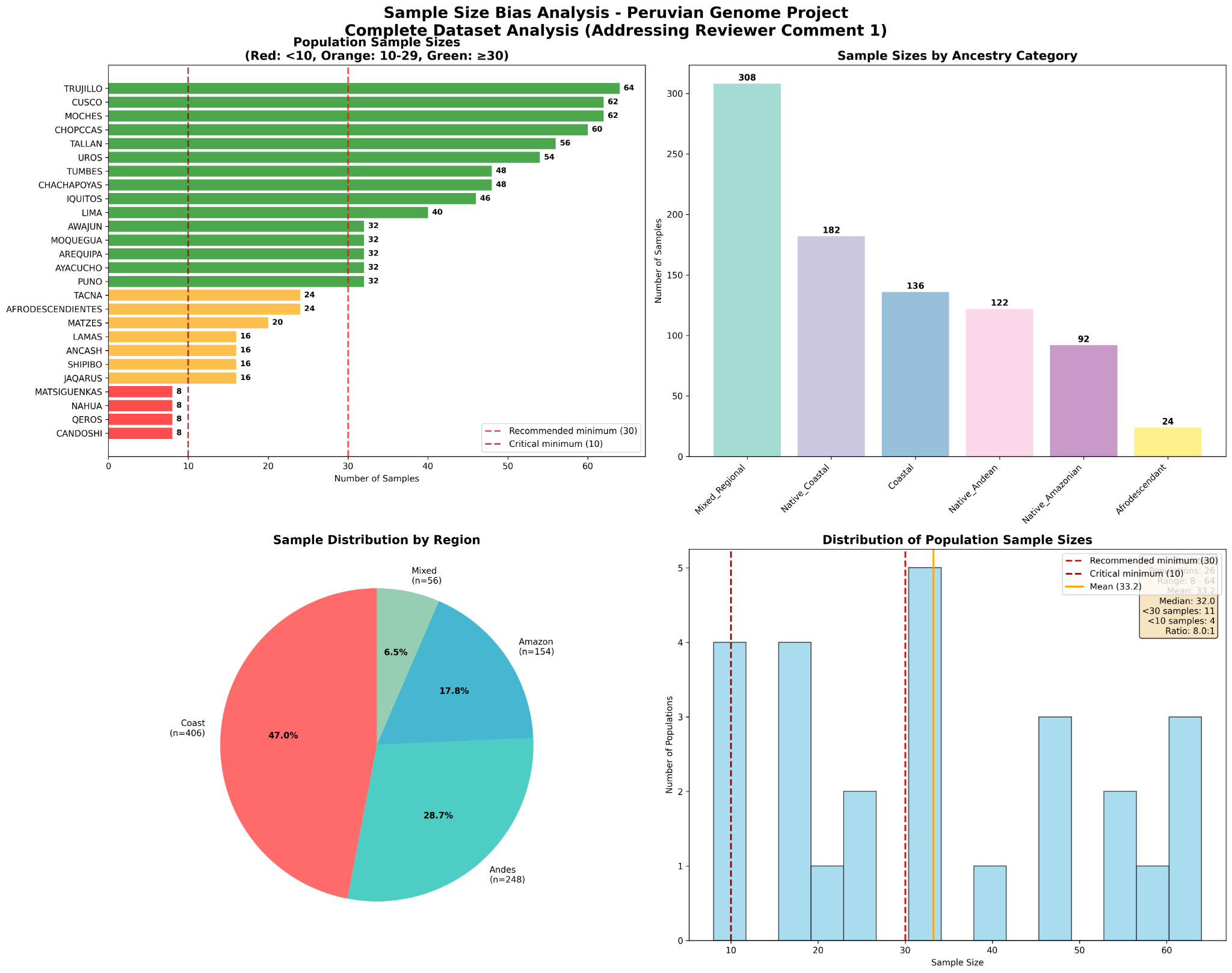


Horizontal bar chart showing sample counts by population. Colors indicate representation thresholds: green (≥30), orange (10–29), red (<10). Dashed lines mark cutoff points.

## Supplementary Figure S2


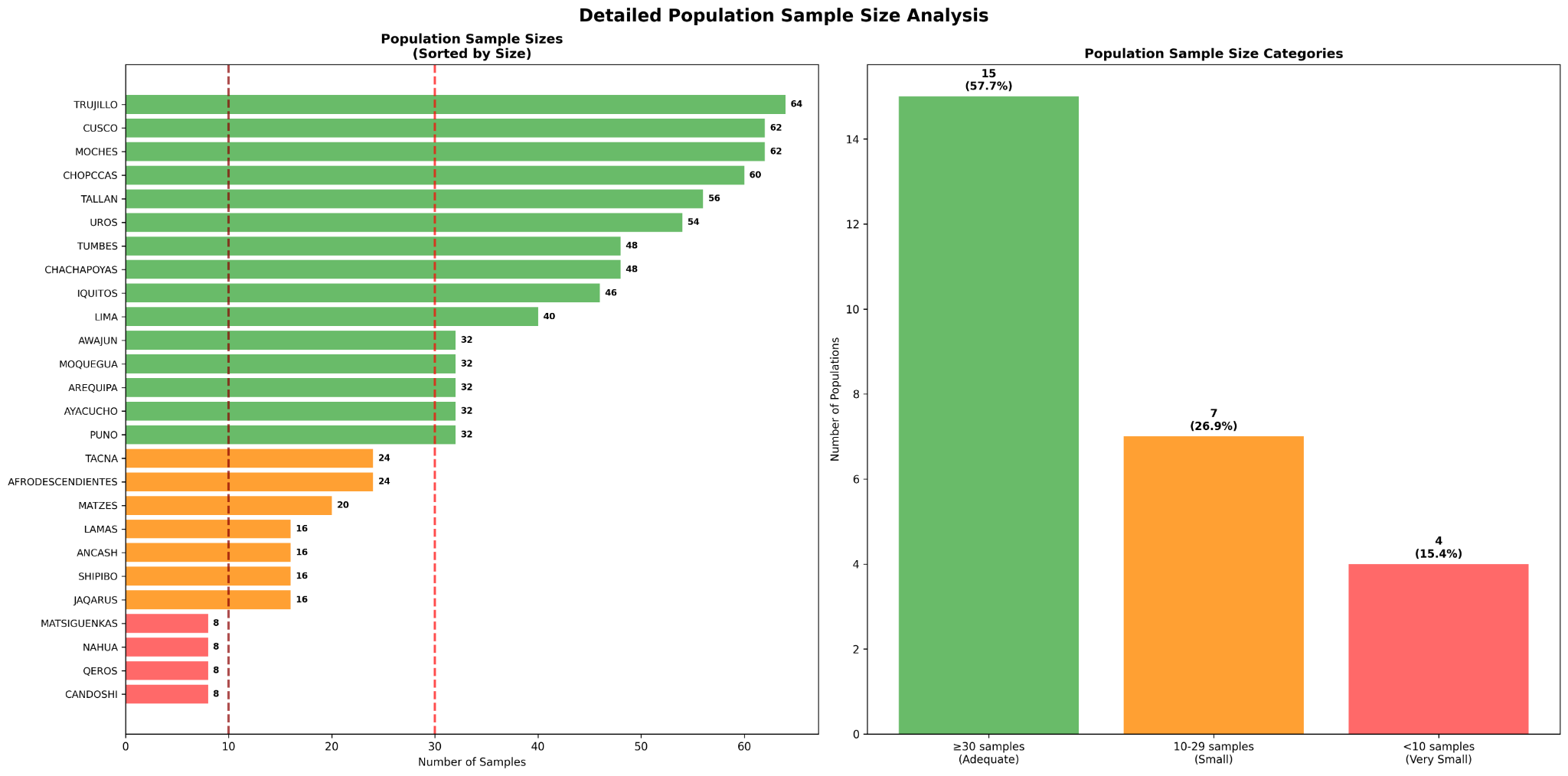


Left panel: sorted bar chart of sample sizes by population. Right panel: bar chart grouping populations into sample size categories with corresponding proportions.

# SNP Chip Ascertainment Bias Analysis

This section provides a comprehensive evaluation of SNP chip ascertainment bias arising from the use of a European-designed genotyping array. We compared genotyping results with whole genome sequencing (WGS) in Native American populations to quantify biases in variant representation, allele frequency spectrum, and chromosomal distribution. Figures S3–S5 summarize key aspects of this analysis.

## Supplementary Figure S3


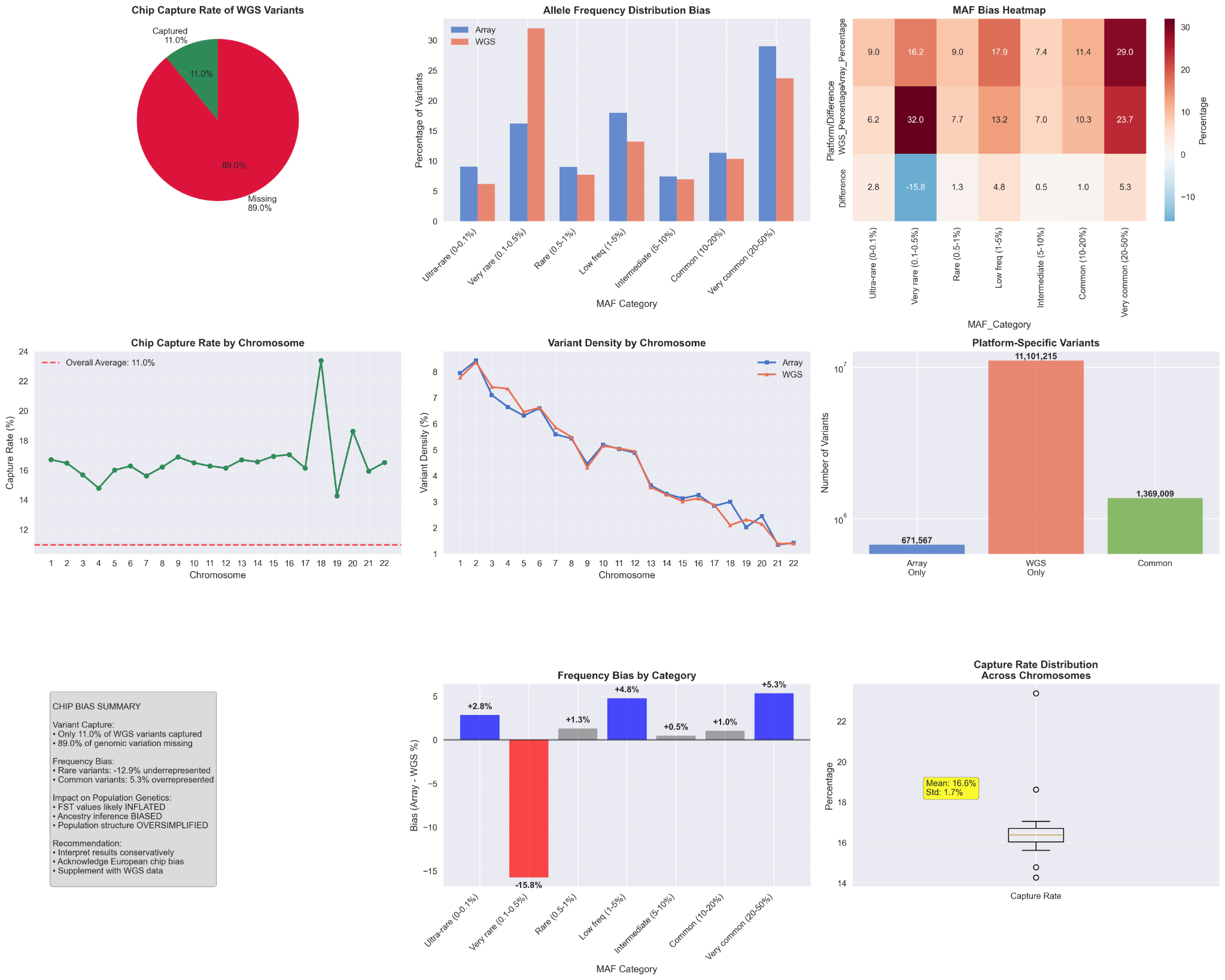


Nine-panel summary comparing variant capture, frequency distributions, chromosomal density, and platform-specific variant counts. Highlights include 11.0% overall capture rate, 11.6% rare variant underrepresentation, and 6.4% common variant overrepresentation.

## Supplementary Figure S4


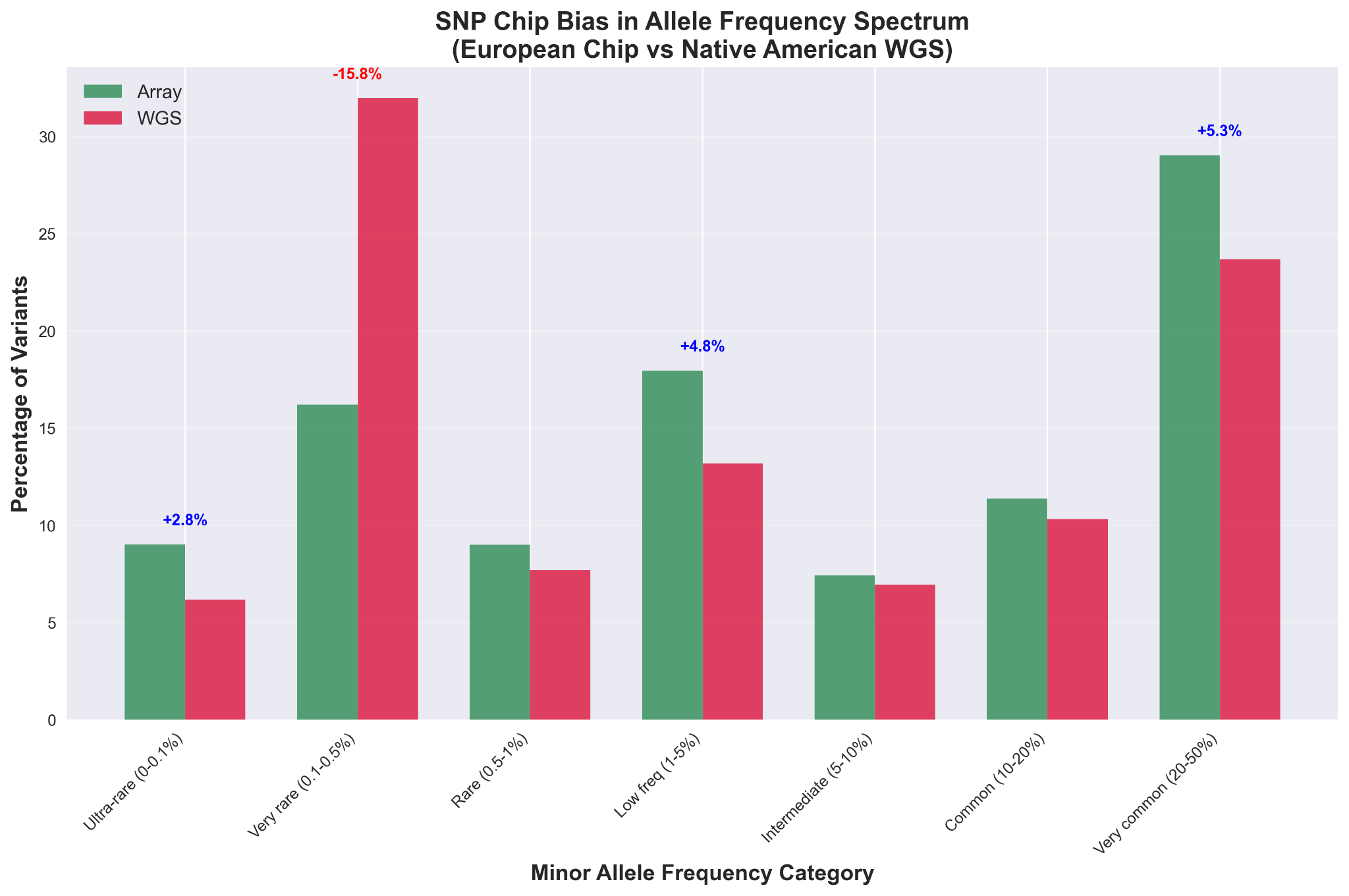


Grouped bar chart comparing allele frequency distributions between SNP array and WGS across minor allele frequency (MAF) bins. Substantial underrepresentation is observed in very rare variants (0.1–0.5% MAF), consistent with known SNP chip design biases.

## Supplementary Figure S5


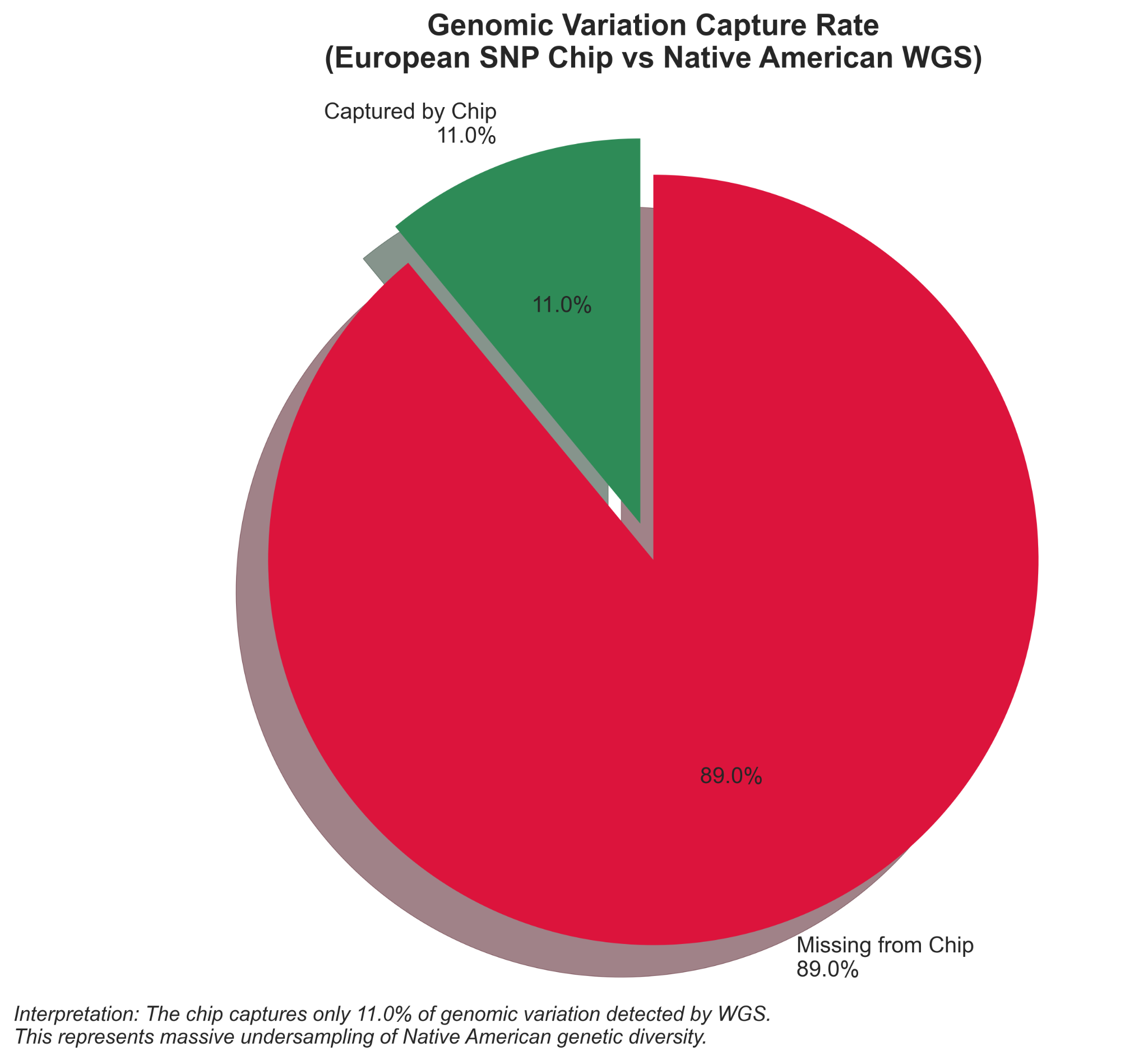


Pie chart illustrating that only 11.0% of genomic variants detected by WGS are captured by the SNP chip. This represents massive undersampling of genomic diversity in Native American populations and highlights the need for WGS-based or ancestry-matched approaches.

# Variant Validation Summary

This figure summarizes the validation of variant-level data quality using VEP and bcftools-derived metrics. We assessed the transition/transversion (Ts/Tv) ratio, estimated a conservative sequencing error rate, and evaluated population-scale evidence supporting the authenticity of detected variants. Figure S6 provides a visual synthesis of these metrics, demonstrating that the dataset meets high standards of genomic quality.

## Supplementary Figure S6


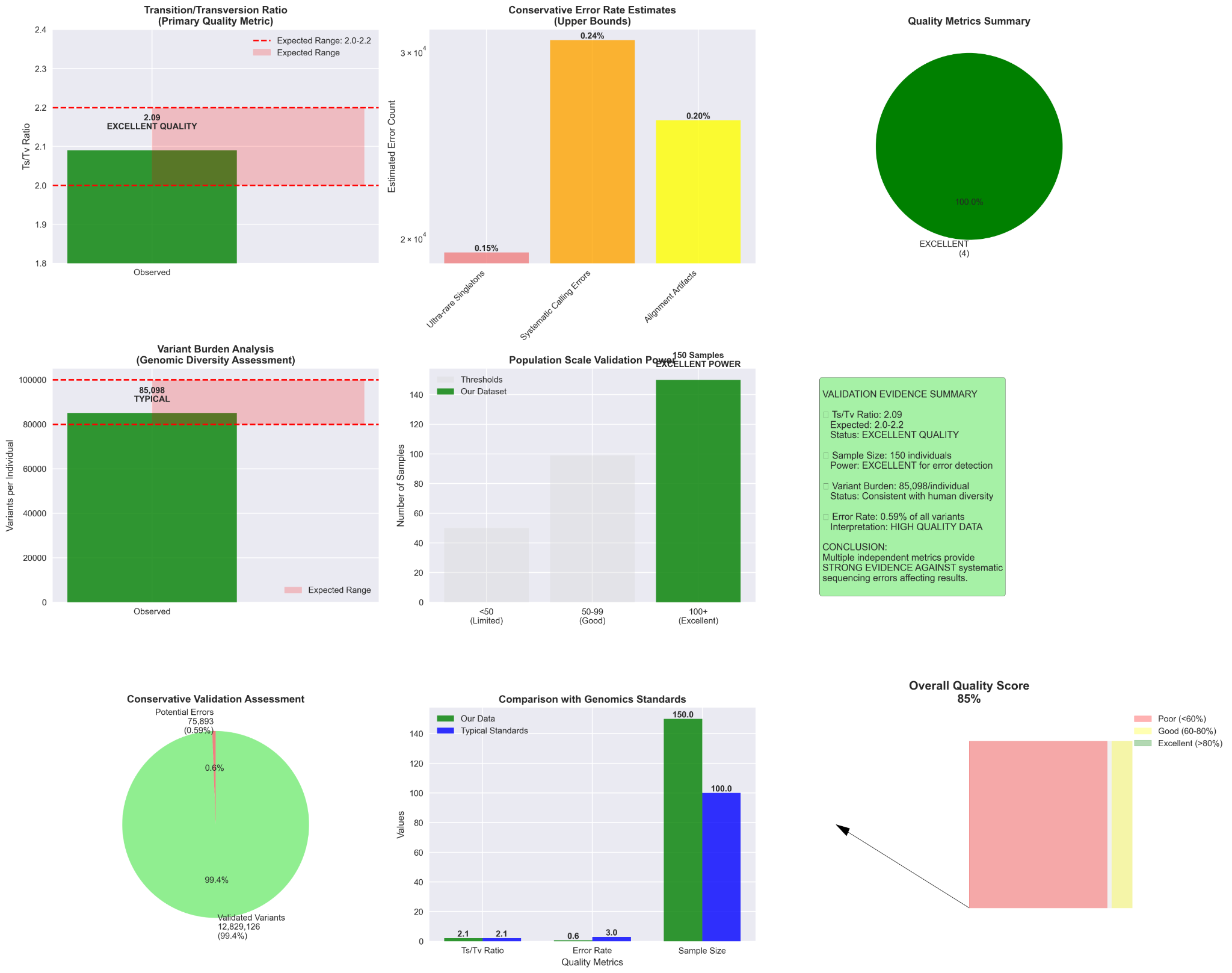


Multi-panel visual summary showing validation of 12.9 million SNPs across 150 Peruvian individuals. Key metrics include a transition/transversion ratio of 2.09, ~85,000 variants per individual, and a conservative error estimate of 0.59%. Indicators of high-quality data include a low multiallelic site rate (0.059%) and sample-scale validation power. These findings support the conclusion that >99% of observed variants represent genuine genetic variation.

# Basic Genomics Quality Control

This figure summarizes the results of standard quality control (QC) analyses performed on both SNP array and whole genome sequencing (WGS) datasets. QC metrics include sample call rate, heterozygosity, variant call rate, minor allele frequency (MAF) distribution, Hardy-Weinberg equilibrium, and sample overlap. Results confirm high data integrity across platforms, supporting the reliability of downstream analyses.

## Supplementary Figure S7


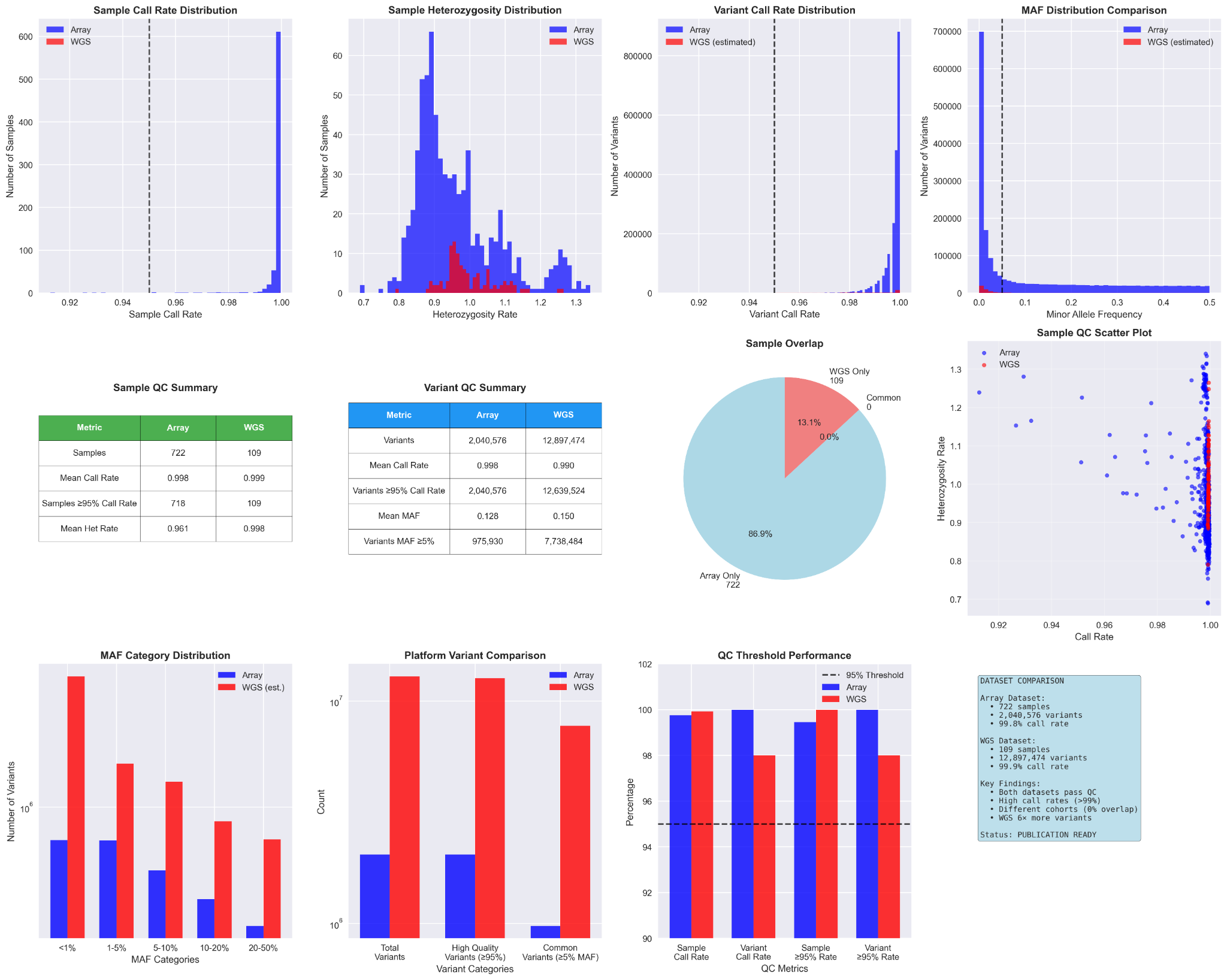


Twelve-panel figure comparing SNP array (n = 722) and WGS (n = 109) datasets across key quality control metrics. All samples met the standard call rate threshold (≥95%), with mean sample call rates exceeding 99% for both datasets. Heterozygosity, MAF distribution, and Hardy-Weinberg equilibrium statistics fall within expected parameters. No overlapping samples were detected, consistent with platform-specific cohorts. These findings confirm that both datasets are of publication-grade quality and suitable for high-confidence genomic analyses.
